# Impacts of Climate Change on the Distribution of Suitable Habitats and Ecological Niche for *Trollius* Wildflowers in Ili River Valley, Tacheng, Altay Prefecture

**DOI:** 10.3390/plants13131752

**Published:** 2024-06-25

**Authors:** Wenhao Fan, Yanyun Luo

**Affiliations:** School of Architecture and Environment, Sichuan University, Chengdu 610065, China; fanwenhao@stu.scu.edu.cn

**Keywords:** *Trollius* wildflowers, climate change, suitable habitat, niche differentiation, mountain flower landscape, plant conservation

## Abstract

Xinjiang in China is distinguished by its distinctive regional landscape and high ecological sensitivity. *Trollius* wildflowers represent a unique and iconic element of the mountain flower landscape in Xinjiang. However, their populations are predominantly distributed in mountainous areas, making them susceptible to climate change. Despite this, the impacts of climate change on the distribution of suitable habitats and ecological niche differentiation for *Trollius* wildflowers have rarely been quantified. Consequently, simulations were conducted using the R-optimized MaxEnt model to predict the suitable habitat distribution of *Trollius* wildflowers. This was based on the occurrence data and environmental variables for the four species of *Trollius* (*T. altaicus*, *T. asiaticus*, *T. dschungaricus*, and *T. lilacinus*) that exist in the study area. The simulation was conducted over a period of time, beginning with the past glacial period and extending to the present, and then to the future (2050s, 2070s, and 2090s) under multiple scenarios (SSP1-2.6, SSP3-7.0, SSP5-8.5). The simulation of suitable habitats enabled the measurement of the ecological niche breadth and differentiation. The results demonstrate that the model predictions are precisely accurate, with AUC values exceeding 0.9. Annual mean temperature (Bio1), isothermality (Bio3), and precipitation in the warmest quarter (Bio18) are the dominant climate variables, in addition to vegetation, elevation, and soil factors. The proportion of suitable habitats for *Trollius* wildflowers varies considerably over time, from 0.14% to 70.97%. The majority of habitat loss or gain occurs at the edges of mountains, while stable habitats are concentrated in the core of the mountains. The gravity center of suitable habitats also shifts with spatial transfer, with the shifts mainly occurring in a northeasterly–southwesterly direction. The SSP1-2.6 scenario results in the sustained maintenance of habitats, whereas the SSP3-7.0 and SSP5-8.5 scenarios present challenges to the conservation of habitats. The threshold of ecological niche breadth for *Trollius* wildflowers is subject to fluctuations, while the ecological niche differentiation also varies. The study aims to examine the evolution of the habitat and ecological niche of *Trollius* wildflowers in Xinjiang under climate change. The findings will provide theoretical support for delineating the conservation area, clarify the scope of mountain flower tourism development and protection of mountain flower resources, and promote the sustainable development of ecotourism and effective utilization of territorial space in Xinjiang.

## 1. Introduction

Plants are integral components of terrestrial ecosystems [1]. Global climate change (GCC) exerts nonlinear, dynamic, and multilevel effects on plant distribution, which in turn alter the structure and function of terrestrial ecosystems. Xinjiang is situated in the hinterland of the Asian–European continent and is a arid or semi-arid region with low precipitation, strong evaporation, relatively sparse vegetation, and fragile ecosystems, which makes it a region of high global ecological sensitivity [2]. *Trollius* is a genus of flowering plants belonging to the *Ranunculaceae* family. It grows mostly in mountains, and four species are produced in Xinjiang: *T. altaicus*, *T. asiaticus*, *T. dschungaricus*, and *T. lilacinus*. The mountain flower landscape formed by them has become a characteristic tourism project in Xinjiang. Recently, due to the sharp rise in emissions caused by human activities, which lead to climate anomalies, *Trollius* wildflowers have become highly susceptible to extinction because of their high climatic sensitivity [3], yet there is a paucity of knowledge regarding the distributional changes and conservation strategies of Trollius wildflowers in Xinjiang in response to GCC.

ITAP (Ili River Valley, Tacheng, Altay Prefecture) is situated in northwestern Xinjiang, with a total area of 325,117.01 km^2^ and the highest biodiversity in Xinjiang [4], making it an ideal location for studying ecosystem heterogeneity in buffering or accelerating the impacts of GCC owing to its climatic sensitivity. ITAP is also the main producing area for *Trollius* wildflowers, and is comprised of three parts. The northern part (Altay) is characterized by a cooler climate and high habitat heterogeneity, with complex flora on Altay Mountain [1,5], and serves as a refuge for numerous endangered and endemic species. The central part (Tacheng) is situated in the west of the Junggar Basin, where the climate is drier in the plains and more humid in the mountains, resulting in the mountainous areas becoming isolated islands for the survival of species [6]. The southern part (Ili River Valley) belongs to western Tien Shan, which is marked by a high degree of species richness and distinctive features, with significant vegetation differentiation, and it is a global biodiversity hotspot [7,8]. Climate change is anticipated to result in increased warming or precipitation in ITAP in the future, which may present challenges to biodiversity conservation.

Previous studies on *Trollius* have mostly concentrated on its medical properties. The efficacy of *Trollius* in clearing heat and detoxifying toxins [9], as well as its antibacterial and anti-inflammatory effects [10], were documented in the *Compendium of Materia Medica*. More recent molecular chemistry research has demonstrated that *Trollius* is rich in flavonoids and alkaloids [11]. Additionally, its medical efficacy in antiviral and antioxidant properties has been confirmed in the context of pharmacology [10,12]. Due to their medical value, the resource of *Trollius* species has been over-harvested anthropogenically and their populations have dwindled dramatically. In the context of plant physiology, scientists have investigated the response of *Trollius* to various environmental stresses, including drought [13], flooding [14], and salinity [15]. Additionally, they have examined the impact of train exhaust emissions and soil heavy metal pollution on its photosynthetic pigment content [16]. Previous studies have paid little attention to the macro-level ecological characteristics and landscape features of *Trollius* wildflowers, but environmental changes have already adversely affected and threatened the habitats of *Trollius* wildflowers. Therefore, it is important to accurately assess the impact of GCC on suitable habitats and ecological niches of *Trollius* wildflowers. This assessment is necessary to protect, develop, and utilize these resources in a rational manner.

The MaxEnt model has been employed with considerable success in the delineation of protected areas for endangered and relict species [17,18], the mapping of invasive species distributions [19,20], crop-designated geographical indications [21,22], and plant disease control [23]. The objective of this study was to determine the intra- and inter-species impacts of GCC on the four species of *Trollius* in ITAP. To achieve this, we developed separate models for each species based on different distribution points and environmental variables. As herbaceous flowers, *Trollius* have a much lower dispersal capacity than animals and lower immunity to GCC than woody plants. Thus, we attempted to explore the magnitude of suitable habitat changes between species under the premise of overlapping survival zones to determine the tolerance level of species to GCC. In addition, we explored the critical role of the transition zone between the more heterogeneous mountainous areas and the more homogeneous plains in the survival of species. Furthermore, different climate scenarios present distinct pathways for the future protection of species. In this study, we identified the effects of the new scenario SSP3-7.0 on the distribution of *Trollius* wildflowers, which emphasizes land-use change for sustainable development and reduced precipitation [24]. Finally, we attempted to determine the evolutionary potential and dispersal movements of species in the face of GCC impacts through changes in species’ ecological niche breadth and overlap indexes.

This study employed the R-optimized MaxEnt model to analyze the impacts of the last glacial period, current conditions, and future scenarios on suitable habitats and ecological niche dynamics for *Trollius* wildflowers. The study aims to achieve the following scientific objectives: (1) to obtain the potential distribution range of *Trollius* under a baseline climate, the most suitable distribution area, and the key factors affecting its distribution; (2) to clarify the impact of GCC on the suitable distribution range and spatial patterns of *Trollius*; and (3) to determine the degree of ecological differentiation of *Trollius* under climate change. The study aims to scientifically delineate the scope of development and protection of *Trollius* wildflower resources and realize the high-quality development of territorial resources.

## 2. Results

### 2.1. Model Accuracy and Dominant Environmental Factors

The results showed that the AUC values were 0.908 for *T. altaicus*, 0.939 for *T. asiaticus*, 0.926 for *T. dschungaricus*, and 0.954 for *T. lilacinus*, indicating that the model prediction accuracy is very high and can be used to simulate the periods of suitable *Trollius* wildflower habitat distribution.

We accumulated the environmental variables with a sum of contributions greater than 80%, and the results indicated that the climatic factors affecting *Trollius* were primarily Bio18, Bio3, Bio4, Bio1, Bio19, and Bio15 (Table 1). First, the largest contributor was Bio18, which contributed 43.2% to *T. altaicus* and 19.0% to *T. lilacinus*. This was followed by Bio3 and Bio4, with Bio3 mainly affecting *T. aisaticus* and Bio4 affecting the distribution of all four species, with a contribution rate ranging from 4.8% to 14.0%. These results indicated that the four species are more sensitive to seasonal changes in temperature. Furthermore, the NDVI influenced the distribution of all four species, with contribution rates ranging from 4.2% to 24.4%. This was particularly evident in *T. altaicus* and *T. dschungaricus*. *T. lilacinus* and *T. dschungaricus* exhibited a strong sensitivity to soil pH, with both species thriving in humus-rich, slightly acidic sandy loam. However, excessive acidity or alkalinity can lead to plant death.

Species distribution thresholds were selected based on distribution probabilities exceeding 50%, then the means were then calculated. *T. altaicus* exhibited a lower Bio18 requirement with a mean of 125.76 mm, while *T. lilacinus* demonstrated a higher requirement for the Bio18 factor with a higher upper threshold limit of 281.20 mm. The difference in the means of the four *Trollius* species for the Bio4 requirement was relatively minor, ranging from 937.17 to 1139.31, with the upper threshold of *T. altaicus* being higher. Furthermore, *T. asiaticus* was more sensitive to the precipitation of the driest month and quarter (Bio14 and Bio17).

### 2.2. Current Distribution of Suitable Habitat for Trollius Wildflowers

**Figure 1:**
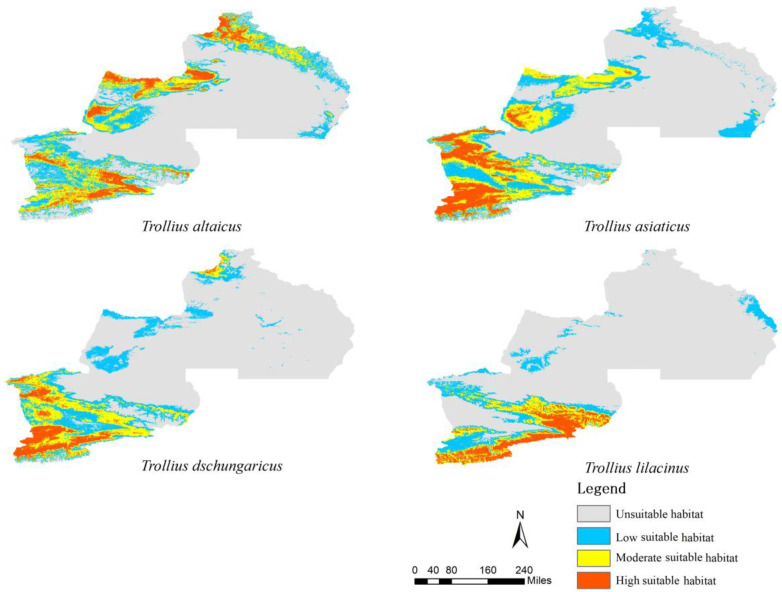
Map of suitable habitat distribution for the four *Trollius* species in the current era.

The results of the simulation indicated that the total area of suitable habitats for *Trollius* wildflowers in ITAP exhibited considerable variation (Table 2). *T. altaicus* displayed the largest total area of suitable habitats, accounting for 40.68%, while *T. lilacinus* showed the smallest total area of suitable habitats, accounting for 20.46%. In the highly suitable habitats, *T. asiaticus* accounted for the largest percentage (8.25%), with a concentration in western Tien Shan in Ili River Valley, the Alatau Mountain in Bole, and the southwestern foothills of the Baluk Mountains in Tacheng (Figure 1). *T. altaicus* accounted for the second-largest percentage (7.61%), with a distribution concentrated in the southern and middle branches of Tien Shan, as well as in the Xialshili and Sayram Lake areas. The Talbahadai Mountains, the Urgarsar Mountains, the Saighur Mountains, the Baluk Mountains, and the northern Altay Mountains, all of which were highly suitable habitats for *T. altaicus*, moderately suitable habitats, and lowly suitable habitats, were identified on the edges of the patches of highly suitable habitats.

The smallest proportion of highly suitable habitat was observed in *T. dschungaricus* (5.12%), which was mainly distributed in western Tien Shan in Ili. Additionally, sporadic distributions of highly suitable habitats were observed in northwest Altay Prefecture (Baihaba scenic area). However, no distribution of moderate or highly suitable habitats for *T. dschungaricus* was observed in Tacheng. In contrast, the highly suitable habitat of *T. lilacinus* was exclusively distributed in western Tien Shan in the Ili River Valley, while only lowly suitable habitats were distributed in Tacheng and Altay Prefecture.

### 2.3. Impacts of GCC on Area Alteration in Suitable Habitats for Trollius Wildflowers

**Figure 2:**
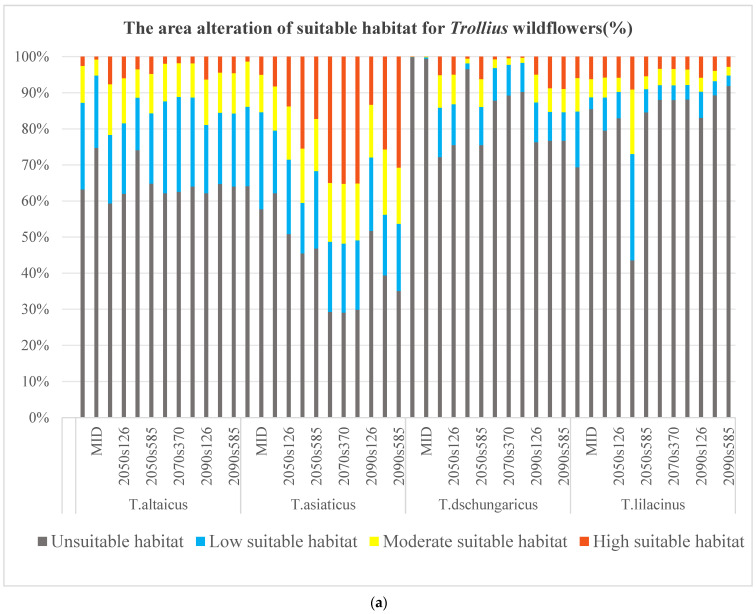

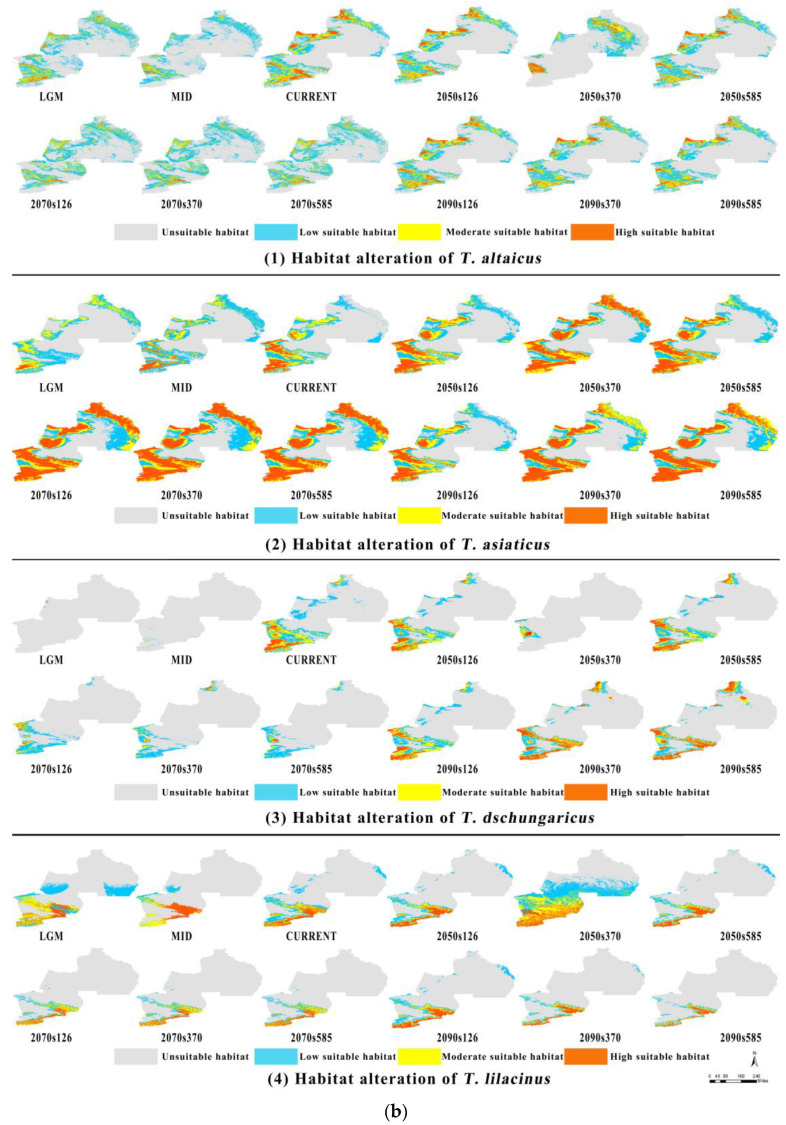
(**a**) Bar chart of suitable habitat alteration for the four *Trollius* species from the last glacial epoch to the current, then to the future. (**b**) Map of suitable habitat alteration for the four *Trollius* species in different periods. Note: 2050s126 represents the distribution of suitable habitats under the SSP1-2.6 scenario in the 2050s.

The alterations in the suitable habitats of the *Trollius* wildflowers exhibited a notable discrepancy from the past to future periods, largely due to the climatic sensitivity of mountainous regions and the habitat preferences of species. Specifically, the totally suitable habitat percentage of the four *Trollius* species varied considerably, ranging from 0.14% to 70.97% (Figure 2a). The smallest change in totally suitable habitat was observed in *T. altaicus*, ranging from 25.27% to 40.68%. In contrast, the largest variation was observed in *T. lilacinus*, ranging from 8.16% to 52.42%. Western Tien Shan and the Altay Mountains were identified as the most comprehensive refuges for *Trollius* wildflowers, while the distribution in other regions varied according to climate conditions (Figure 2b).

The Last Glacial Maximum (LGM) was a period of transition from a cold to a warm Earth, with average temperatures 5 to 10 °C lower than the current era. However, the average temperature in the Middle Holocene (MID) was 3–5 °C higher. From the LGM to the current era, the areas of highly suitable habitats for the four *Trollius* species showed an upward trend, while the areas of moderate and lowly suitable habitats were relatively fluctuating. It was evident that the totally suitable habitats of *T. altaicus* and *T. lilacinus* exhibited a decline and subsequent increase. Both species exhibited low values in the MID, indicating that they are more sensitive to warming. The totally suitable habitat of *T. asiaticus* increased and then decreased, while *T. dschungaricus* exhibited a nearly absent distribution of suitable habitat during the glacial era.

The greatest proportion of total and highly suitable habitats was exhibited by *T. altaicus* in the SSP1-2.6 scenario, while the lowest proportion was observed in the SSP3-7.0 scenario. In the 2050s, *T. altaicus* exhibits a declining trend in totally suitable habitat, as evidenced by a reduction in highly and moderately suitable habitats and fluctuations in lowly suitable habitats. The percentage of totally suitable habitats under the three scenarios for the 2070s remains relatively consistent, with the SSP1-2.6 scenario still exhibiting the highest percentage of totally and highly suitable habitats (37.85% and 1.93%). The percentage of highly suitable habitats under the three scenarios falls below 2% in the 2070s, representing a significant decline from the current era (7.61%). However, by the 2090s, the percentage of highly suitable habitats rebounds to more than 4%, with the percentage of highly suitable habitats under the SSP1-2.6 scenario rising to 6.33%.

For *T. asiaticus*, it is predicted that the area of both moderately and highly suitable habitats will increase. In the 2050s, the area of moderately suitable habitats increases to over 14% in three scenarios, exceeding the current level. In contrast, the proportion of lowly suitable habitats decreases to 13.99% under the SSP3-7.0 scenario, while the proportion of highly suitable habitats increased to 25.46%. In the 2070s, there is less difference between the classes of suitable habitats. However, the percentage of highly suitable habitats exceeds 35%. Totally suitable habitats exceed 70%, making this the most widely distributed period for *T. asiaticus.* In the 2090s, totally suitable habitats for *T. asiaticus* decrease to 48.27% under the SSP1-2.6 scenario, yet remain higher than the 37.83% observed at present.

For *T. dschungaricus*, the proportion of totally suitable habitats exhibits a decline in the 2050s and 2070s, followed by a recovery in the 2090s. In the 2050s, the habitat conditions of *T. dschungaricus* demonstrate a high degree of maladaptation to the SSP3-7.0 scenario, with only 3.45% of the totally suitable habitats under the scenario and a distribution confined to the Ili River Valley. In the 2070s, the proportion of highly suitable habitats under all three scenarios drops below 1%, with the species confined to western Tien Shan and the Altay Mountains. By the 2090s, however, the proportion of highly suitable habitats rebounds to more than 23%, with the SSP1-2.6 scenario displaying the greatest total area of suitable habitats, yet the smallest proportion of highly suitable habitats and lower than the proportions of highly suitable habitats under the SSP3-7.0 and SSP5-8.5 scenarios.

The suitable habitats for *T. lilacinus* will undergo a decline in the future. By the 2070s, the proportion of highly suitable habitats declines to approximately 3%. The highest proportion of highly suitable habitats is observed in SSP5-8.5, but the lowest proportion of totally suitable habitats is also evident. In the 2090s, the greatest proportion of totally suitable habitats and all classes of suitable habitat are exhibited under the SSP1-2.6 scenario, with a rebound from the 2070s SSP1-2.6 scenario. Nevertheless, the proportion of all classes of suitable habitat in the SSP3-7.0 and SSP5-8.5 scenarios continue to decline in comparison to the 2070s.

### 2.4. Impacts of GCC on Spatial Transfer of Suitable Habitats for Trollius Wildflowers

**Figure 3:**
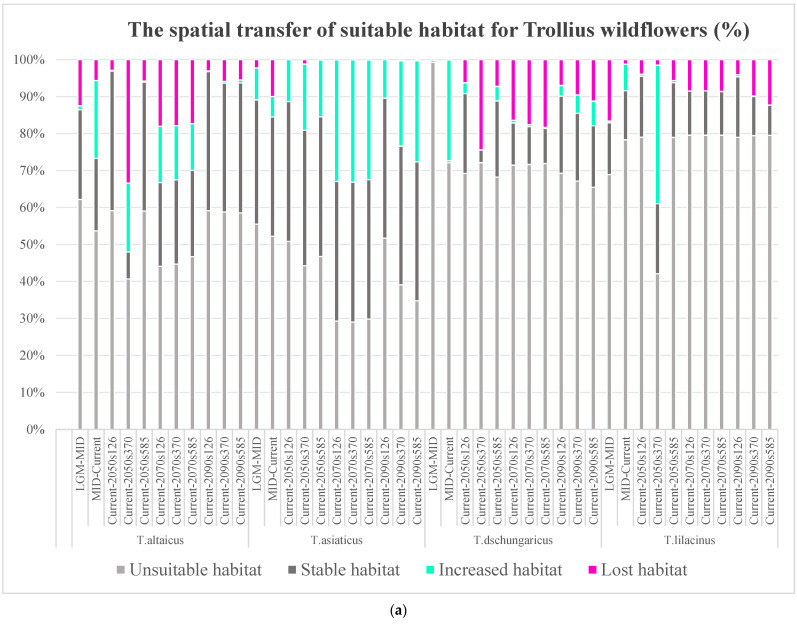

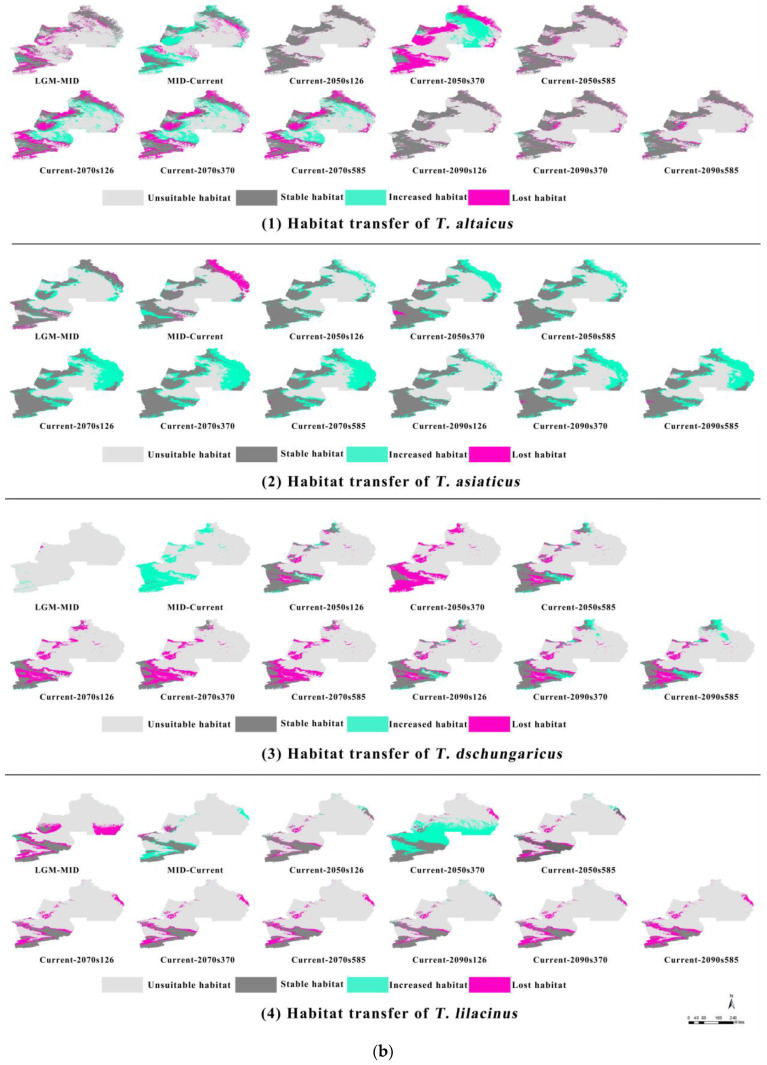
(**a**) Bar chart of suitable habitat spatial transfer for the four *Trollius* species from the last glacial epoch to the current, then to the future. (**b**) Map of suitable habitat spatial transfer for the four *Trollius* species between different periods.

The result of the spatial transfer of suitable habitats indicates that the edges of habitat patches are susceptible to GCC. They are also the main areas where habitat is added or lost (Figure 3b). Habitat additions and losses varied among species. *T. dschungaricus* and *T. lilacinus* had the lowest average habitat additions, while *T. asiaticus* had the most habitat additions. Habitat changes in *T. altaicus* were relatively fluctuating.

For *T. altaicus*, the extent of its habitat has diminished by 12.50% during the LGM–MID period. This indicates that warmer temperatures have a significant impact on alpine flowers. During the MID to the current period, the extent of suitable habitats increased by 21.07%, with the majority of this recovery occurring in mountainous regions. By the 2050s, the spatial changes in suitable habitats are not apparent in the SSP1-2.6 or SSP5-8.5 scenarios. In contrast, in the SSP3-7.0 scenario, there is a dramatic shift in habitats. The habitats lost in the 2070s represent approximately 17% to 18% of the total, with the majority of these losses occurring in the higher-elevation areas of the mountains. The 2090s are characterized by a dominance of stable habitats, with a mere 1% of increased habitats and a loss of habitats amounting to 3% to 5% (Figure 3a).

For *T. asiaticus*, habitat changes were dominated by loss. The increased habitats during the LGM–MID period were primarily situated on the edges of mountainous areas, where the warmth and light were more conducive to harvesting. In the MID–current period, a significant expansion in habitats occurred in the Ili River Valley, with a subsequent contraction in Altay. From the current period to the 2050s, the proportion of lost habitats ranges from 0.01% to 1.21%. Conversely, there was a notable increase in the number of added habitats, which occurred mainly in the Altay Mountains and northern Tien Shan. By the 2070s, the proportion of lost habitats ranges from 0.01% to 0.08%, while the increased habitats are distributed across a range of areas, with the largest patches located in Altay. By the 2090s, the proportion of lost habitats ranges from 0 to 0.32%, with the greatest increase occurring under the SSP5-8.5 scenario.

For *T. dschungaricus*, habitat changes are dominated by additions. The percentage of non-habitat during the LGM–MID period was 99.26%, while the percentage of increased habitats in the MID–current period was 27.32%, which was concentrated in mountainous areas. During the current–2050s period, the largest percentage of lost habitats (24.42%) and the lowest percentage of increased habitats (0.02%) are observed under SSP3-7.0. By the 2070s, the number of lost habitats continues to rise, with the majority of these habitats located in the low-elevation areas of the Ili River Valley. By the 2090s, the greatest increased habitats are observed in SSP5-8.5. However, this scenario still had the highest number of lost habitats, in contrast to the SSP1-2.6 scenario, which was more stable.

For *T. lilacinus*, suitable habitats declined by 16.63% during the LGM–MID period and increased by 85.47% during the MID–current period. From the current to the 2050s, the increased habitats under the SSP3-7.0 rise by 37.46%, with the majority concentrated in the Ili River Valley and the Junggar Basin. The remaining two scenarios exhibited a similarly low percentage of increased habitats, with less than 1%. By the 2070s, the increased habitats are all 0, while the lost habitats constitute approximately 8%. The lost habitats are primarily located on the edge of western Tien Shan. By the 2090s, the SSP1-2.6 scenario exhibits the greatest increase in habitats, at 0.58%. This is primarily observed in the Alatau Mountains, the Xialshili area, the Saighur Mountains, and the Altay Mountains. In contrast, the SSP5-8.5 scenario exhibits the fewest new additions and the most lost habitats.

### 2.5. Impacts of GCC on Gravity Center of Suitable Habitats for Trollius Wildflowers

**Figure 4:**
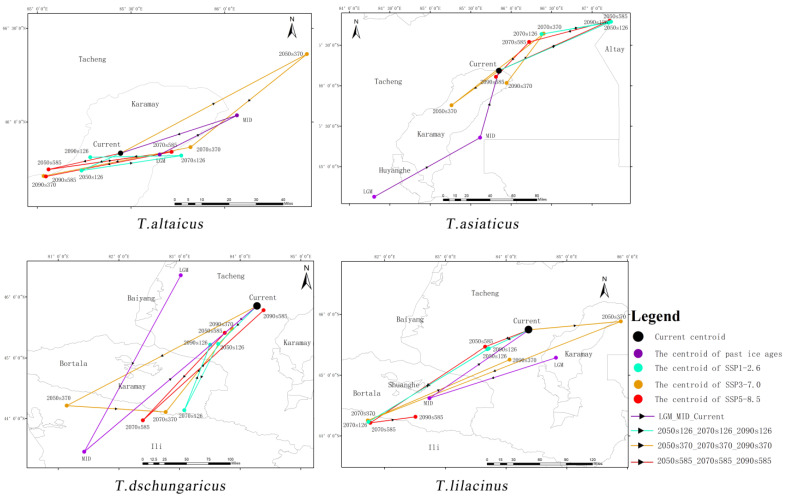
The gravity center migration of suitable habitats for the four *Trollius* species from the last glacial epoch to the current, then to the future.

For *T. altaicus*, overall, the gravity center shifted to low latitudes (Figure 4). The direction of the gravity center of suitable habitats exhibited a notable shift, with a predominant northeast–southwest trajectory. During the LGM–MID period, the suitable habitats in the southern Tacheng experienced a substantial reduction, prompting a northward shift in the gravity center of *T. altaicus* by 39.47 km. However, in the MID–current period, the suitable habitats in southern Tacheng demonstrated a notable recuperation, resulting in a return of the gravity center to its original position. In the SSP1-2.6 and SSP5-8.5 scenarios, shorter migration distances and less spatial variability of the gravity center were observed. In the SSP3-7.0, the gravity center exhibited a significant shift of 96.64 km to high latitudes to avoid the intense heat. However, by the 2070s, the gravity center shifts southwest by 73.08 km, and by the 2090s, it shifts further, reaching 63.17 km to the southwest (Table S3).

The gravity center of *T. asiaticus* shifted from the southern to the northern part of Karamay during the LGM–MID–current period. In the SSP1-2.6 scenario, the expansion of habitats was primarily observed in the Sagur Mountains and the Altay Mountains. Consequently, the gravity center shifted to higher latitudes by 124.25 km in the 2050s. In the 2070s and 2090s, the gravity center exhibits a meandering shift from southwest to northeast. In the SSP3-7.0 scenario, the gravity center undergoes a southwest–northeast–southwest shift with a shorter distance. In contrast, in the SSP5-8.5 scenario, the gravity center undergoes a sharp shift to the northwest by 125.51 km in the 2050s, followed by a return to the current gravity center after two migrations from the 2070s to the 2090s.

The migration of the gravity center of suitable habitats for *T. dschungaricus* exhibited fluctuations and a meandering pattern. During the LGM–MID–current period, the gravity center initially migrated from Tacheng to Ili River Valley and subsequently reverted to eastern Tacheng. In the SSP1-2.6 scenario, the gravity center initially shifts 220.14 km to the southwest, followed by a 124.60 km shift back to the northeast in the 2090s. In the SSP3-7.0 scenario, the increased suitable habitats occur exclusively in the Ili River Valley during the 2050s, and consequently, the gravity center shifts abruptly 310.67 km to the Ili River Valley. There is a restoration of suitable habitats in the mountainous regions in the eastern part of the Ili River Valley during the 2070s, resulting in a further shift in the gravity center by 310.67 km eastward. In the SSP5-8.5 scenario, the gravity center migrates 67.52 km southwest in the 2050s, and the extent of degradation of suitable habitats in Altay and Tacheng is considerable in the 2070s, with the gravity center shifting southward to lower latitudes. In the 2090s, suitable habitats in Altay and Tacheng experience a recovery, resulting in the gravity center returning to a position near its original location.

For *T. lilacinus*, during the LGM–MID–current period, the gravity center of suitable habitats for *T. lilacinus* shifted southwest from Karamay to Bole by a distance of 180.76 km, and then moved northwest to the central part of Tacheng. In the future SSP1-2.6 scenario, the gravity center moves 65.66 km southwest in the 2050s and continues to move southwest to Ili River Valley in the 2070s by a distance of 202.99 km. In the 2090s, it moves back to the area where the 2050s center is located. In the SSP3-7.0 scenario, the gravity center shifts 117.64 km to the northeast in the 2050s, migrates 373.55 km to the southwest of Ili River Valley in the 2070s, and travels 214.13 km back to the northwest in the 2090s. In the SSP5-8.5 scenario, the gravity center is initially displaced slightly to the southwest by 64.61 km in the 2050s, subsequently continuing to migrate southward to Ili River Valley in the 2070s, and finally moving to the south of Bole in the 2090s.

### 2.6. Impacts of GCC on Ecological Niche Breadth, Overlap, and Equivalency for Trollius Wildflowers

**Figure 5:**
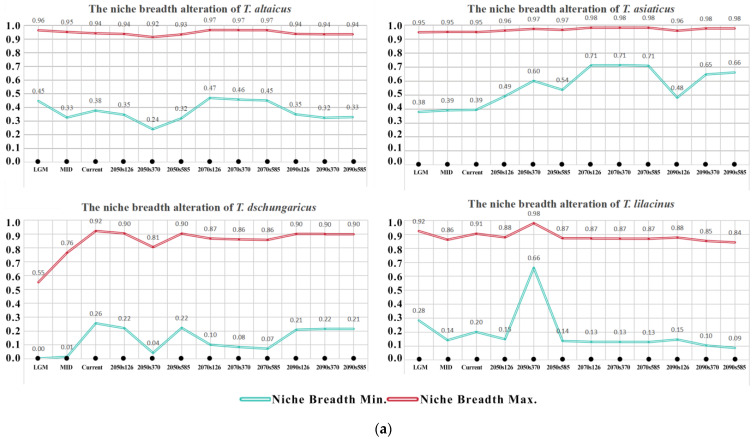

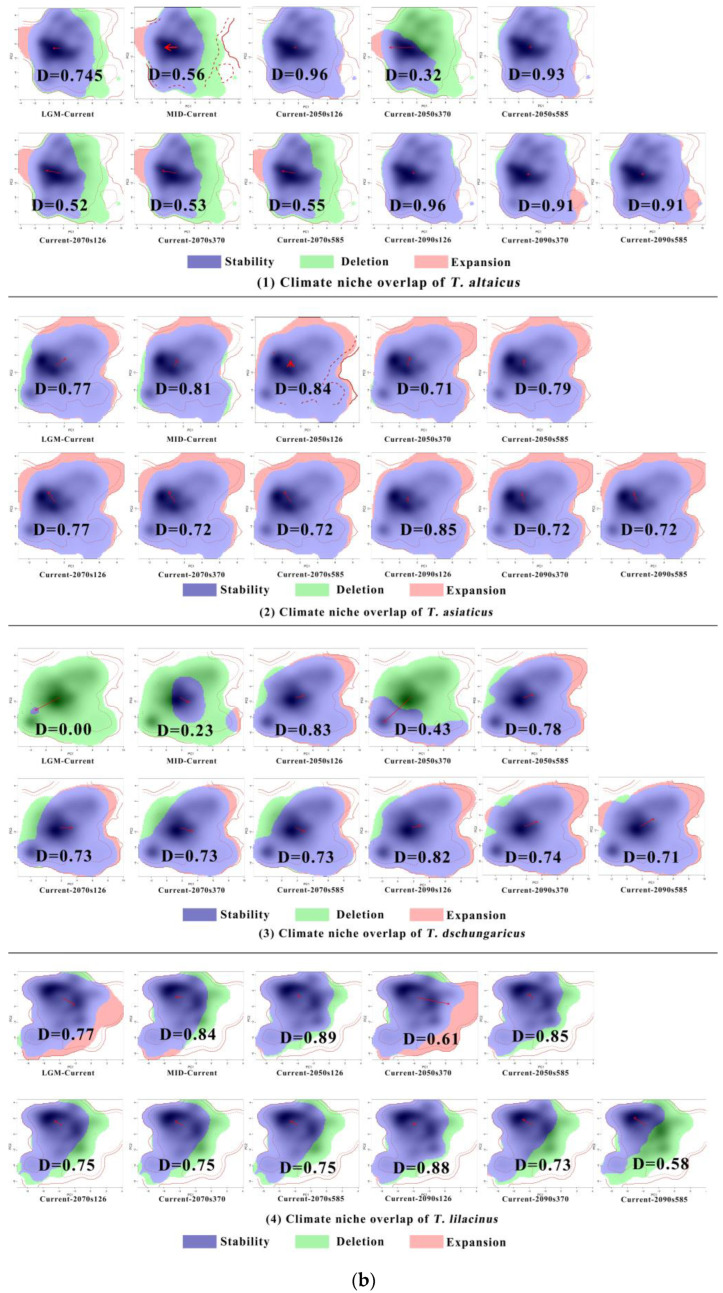
(**a**) Alteration in climate niche breadth for the four *Trollius* species. (**b**) Climate niche overlap of four *Trollius* species. Note: The solid and dashed contour lines illustrate 100% and 50% of the available environmental space, respectively. Red arrows show how the center of the climatic niche (solid line) and the background range (dashed line) of *Trollius* moved between the two ranges. D = 0.888 indicates that the Schoener’s D niche overlap index is 0.888. The *x*-axis and *y*-axis represent the value of principal components (PC1 and PC2).

The maximum threshold of niche breadth for *T. altaicus* and *T. asiaticus* was consistently above 0.9 in each period, while the minimum threshold exhibited fluctuations (Figure 5a). The minimum threshold of niche breadth for *T. altaicus* exhibited a “falling–rising–falling” pattern, with a minimum value of 0.24 reached in the 2050s under the SSP3-7.0 scenario. Conversely, the minimum threshold of niche breadth for *T. asiaticus* exhibited a continuous increase, reaching a peak in the 2070s, followed by a decrease in the 2090s, and finally reaching a minimum value of 0.48 in the 2090s under the SSP1-2.6 scenario. The maximum and minimum threshold changes in niche breadth of *T. dschungaricus* both exhibited a similar wave-like pattern, with peaks of 0.92 and 0.26 observed in the present and troughs of 0.55 and 0.00 observed during the LGM, respectively. The niche breadth of *T. lilacinus* exhibited greater stability, although it displayed a pronounced peak in the 2050s under the SSP3-7.0 scenario, due to the rapid expansion in suitable habitats for *T. lilacinus* in this scenario.

The results of principal component analysis (PCA) indicated that PCA (PC1 and PC2) based on screened climatic variables explained 84.63% of the climatic variance at the distribution site of *T. altaicus*, 81.50% of *T. asiaticus*, 81.70% of *T. dschungaricus*, and 86.00% of *T. lilacinus.* In particular, temperature and precipitation were found to influence the geographic distribution and ecological niche variation of *Trollius* wildflowers (Figure S2).

The results of the climatic ecological niche overlap dynamics and equivalency test, based on species distribution data and climatic variables, revealed that the degree of climatic ecological niche differentiation of the four *Trollius* species varied greatly (Figure 5b). For *T. altaicus*, comparing past with present niches, there was a differentiation characterized by deletion. In the future, except for the SSP3-7.0 scenario in the 2050s and all scenarios in the 2070s, the overlap indexes for rest periods and scenarios were above 0.9 and essentially undifferentiated [25]. With regard to *T. asiaticus*, the differentiation of ecological niches in the future was mainly dominated by expansion, which was attributed to the substantial growth in suitable habitats. In contrast, *T. dschungaricus* was distributed scarcely in the past glacial period, and thus there was a large deletion in the ecological niche compared to the current. Under the SSP3-7.0 scenario in 2050s, the suitable habitat of *T. dschungaricus* retreated to the Ili River Valley, resulting in a severe ecological niche deletion. However, the overlap index for the remainder of the period was approximately 0.7–0.8, indicating a slight degree of differentiation. The type of ecological niche differentiation observed in *T. lilacinus* was characterized by deletions, which were related to its spatial distribution and its capacity to hold ecological resources at different times.

## 3. Materials and Methods

### 3.1. Species Occurrence and Processing

**Figure 6:**
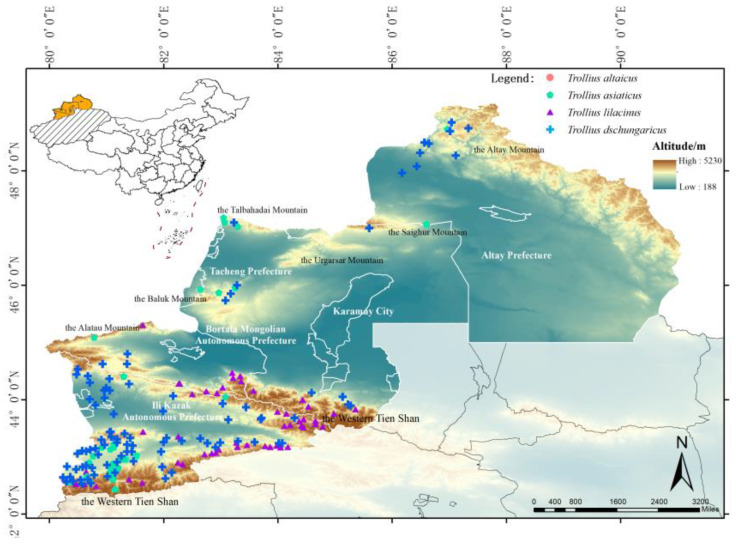
Distribution of locations of the four species of *Trollius* modeled in Ili River Valley, Tacheng, Altay Prefecture (ITAP).

**Figure 7:**
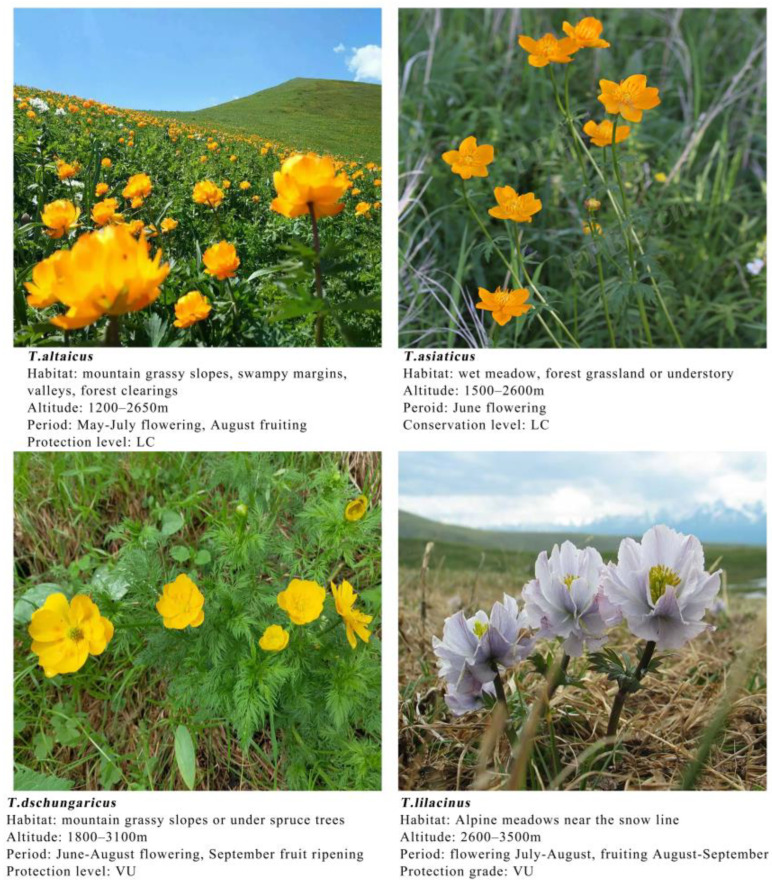
Photographs of four *Trollius* species. Sources: authors, https://www.iplant.cn (accessed on 13 May 2024), and https://zhihu.com (accessed on 13 May 2024) (LC represents least concern, VU represents vulnerable).

The occurrence data of the four *Trollius* species were obtained from the Global Biodiversity Information Facility (https://www.gbif.org/, accessed on 5 June 2023), National Specimen Information Infrastructure (http://nsii.org.cn/, accessed on 16 June 2023), Chinese Virtual Herbarium (https://www.cvh.ac.cn/, accessed on 16 June 2023), Herbarium, Institute of Botany, Chinese Academy of Sciences (http://pe.ibcas.ac.cn/peweb/, accessed on 16 June 2023), and published literature, as well as actual field surveys. For the actual images and county or district records, latitude and longitude data were obtained using the Google Maps pickup coordinate system. A total of 298 occurrences were collected. To reduce the spatial autocorrelation of occurrence data, redundant occurrence data were removed in ENMTools [26]. After screening according to the grid cell image elements, a total of 274 valid occurrence data were obtained, of which 94 were *T. altaicus*, 97 were *T. asiaticus*, 97 were *T. dschungaricus*, and 51 were *T. lilacinus*. The distribution of the four *Trollius* species in the study area and their photographs are shown in Figure 6 and Figure 7, respectively.

### 3.2. Environmental Variables and Processing

The distribution of plants is influenced by a multitude of environmental factors. The environmental factors selected for this study included climate, topography, soil, and vegetation. The climate factors were obtained from the WorldClim database, which includes 19 climate variables for six periods: the last glacial period (the Last Glacial Maximum (LGM) and the Middle Holocene (MID)), the current period (1970–2000), and the future (2050s, 2070s, and 2090s). In selecting the future climate scenarios, the study considered the varying adaptations of different species to extreme climates [27], and thus a set of new emission scenarios driven by different socioeconomic factors published by the IPCC in 2021 was selected, containing SSP1-2.6, SSP3-7.0, and SSP5-8.5. Three pathways represent low, medium, and high levels of radiation pressure and economic development intensity. Elevation data were obtained from the Geospatial Data Cloud (http://www.gscloud.cn/ (accessed on 12 October 2023)). The slope and aspect were calculated in ArcGIS. The vector ruggedness measure (VRM) and terrain roughness index (TRI) were collected from EarthEnv, an open-source database. Soil factors were obtained from the HWSD raster dataset of the World Soil Database (WSD), which is provided by the International Food and Agriculture Organization (IFAO) (https://www.fao.org/soils-portal/ (accessed on 12 October 2023)). The normalized difference vegetation index (NDVI) was acquired from the Center for Resource and Environmental Science and Data (CRESD) (http://www.resdc.cn (accessed on 12 October 2023)). Cropping and resampling were conducted in ArcGIS to ensure that the accuracy was standardized to 30 s.

**Figure 8:**
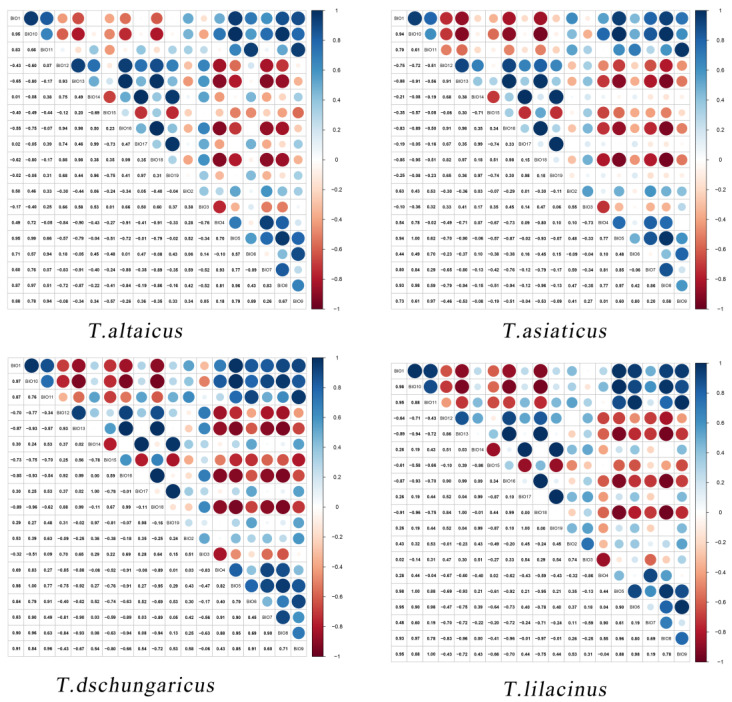
Heat map of climate factor correlations for four *Trollus* species.

To avoid multiple correlations among variables and to account for the role of topography, soil, and vegetation factors in maintaining the habitats of the four *Trollius* species, we conducted correlation analyses for climate variables. First, current climate variables and occurrence data were imported into the MaxEnt model, and pre-experiments were conducted for each species to exclude variables with a contribution rate of 0. Then, Pearson correlation analysis was conducted for climate variables (Figure 8), and those with correlation coefficients |r| < 0.8 were retained, while those with |r| ≥ 0.8 were retained only for those with a higher contribution rate [28]. Climate variables were screened for the four *Trollius* species, and the corresponding climate variables were extracted for modeling (Table 3).

### 3.3. MaxEnt Model Screening and Optimization

Utilizing the default parameters in the MaxEnt model may result in the overfitting of simulation results that are challenging to interpret [29]. To address this issue, we employed the kuenm R package for model parameter calibration and evaluation, utilizing the previously screened distribution data and environmental variables. The accuracy of the MaxEnt model is influenced by two parameters: the feature class (FC) and the regularization multiplier (RM). The FC includes linear (L), quadratic (Q), product (P), hinge (H), and threshold (T). The RM parameter affects the distributional focus of the species, with too small an RM resulting in overfitting and a larger value of RM yielding a wider range of predictions [30]. The value of RM is generally taken in the range [0.1,4]. In this study, the occurrence data for each species were divided into training and test sets using the kuenm_occsplit.R package, and the kuenm_calibration R package was employed to construct candidate model ensembles in conjunction with the environmental variables. An omission rate of less than 5% and a delta AICc value of less than 2 was selected as the optimal parameter combination for constructing the model (Table 4).

### 3.4. Suitable Habitat Simulation of Trollius Wildflowers: Rank Division, Space Transfer, and Gravity Center Migration

The screened occurrence data and environmental variables were entered into MaxEnt v3.4.1. A random 75% of the occurrence data was designated as the training set, while the remaining 25% was set aside as the test set. The parameters were set based on Table 4, and the model was run 10 times. Jackknife was employed to ascertain the contribution of distinct environmental variables to the *Trollius* wildflowers. Receiver operating characteristic (ROC) and area under the curve (AUC) were utilized to assess the accuracy of the model. If the AUC was equal to or greater than 0.9, the prediction accuracy was deemed to be excellent [31].

The simulation results were imported into ArcGIS 10.3, converted to a raster, and classified into four classes using Natural Break: highly suitable habitat, moderately suitable habitat, lowly suitable habitat, and non-habitat. This method was also employed to analyze past and future periods.

The spatial transfer of suitable habitat for *Trollius* wildflowers was analyzed by overlaying the current and future (2050s, 2070s, and 2090s) suitable habitat maps. The binary suitable habitat maps of two periods were overlaid using the Distribution Changes between Binary SDMs tool, resulting in grid values of 0 (unsuitable habitat, both current and future), 1 (lost habitat), 2 (increased habitat), and 3 (unchanged habitat, both current and future).

The reclassification results were processed into binary files using Raster tools in SDM Toolbox v2.5, which determined the gravity centers of the suitable habitats in different periods. The gravity centers of adjacent periods were combined and converted into lines to obtain the direction and distance of the gravity centers’ migration from the last glacial period to the future.

### 3.5. Climate Ecological Niche Changes

The ecological niche is defined as the minimum threshold of habitat required by a species within an ecosystem and can be quantified by the niche breadth [32]. The climate niche overlap of a species can be understood as a reflection of its survival strategy to cope with GCC. ENMTools was employed to quantify the extent of species-level ecological niche breadth changes based on the results of the distribution of suitable habitats for *Trollius* in each period obtained in the previous section, while the ecospat package in R was utilized to assess climate niche alterations. First, principal component analysis (PCA) was conducted, then, based on the observed species distribution density and climate background space, climate density grids were constructed for the current period and the predicted period [33]. The Schoener’s D ecological niche overlap index was calculated by comparing the spatial distribution of ecological niches in two periods.

Schoener’s D ranges between 0 (no overlap) and 1 (>0.6 with overlap), with 1000 iterations performed for each repetition to provide an equivalency assessment [34]. If the observed D is less than the expected value, the hypothesis of equivalency between the climate and the ecological niche is rejected, indicating ecological niche differentiation between the two periods.

## 4. Discussion

**Figure 9:**
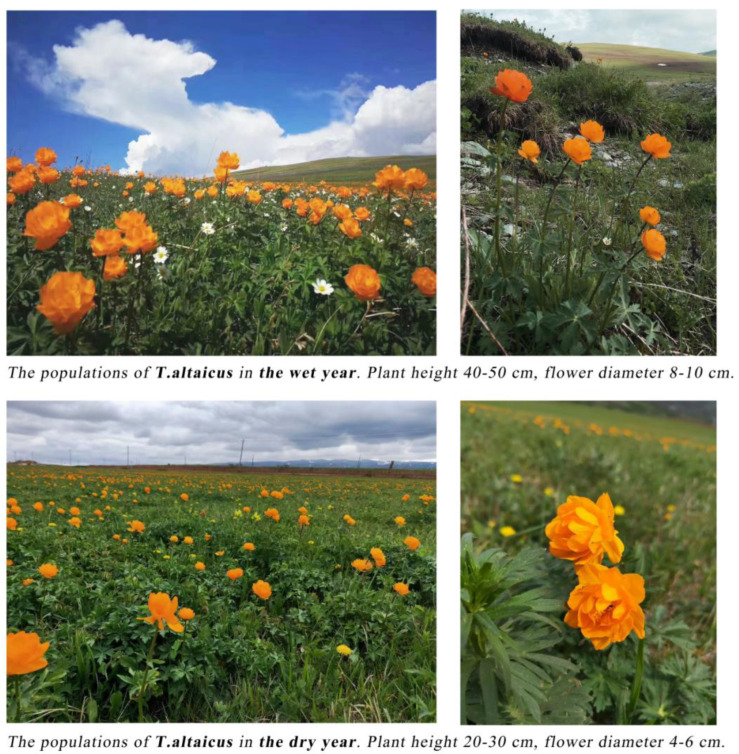
Populations of *Trollus altaicus* in dry and wet years. Image source: authors, Talbahadai Mountain, Tacheng.

*Trollius* wildflowers, with their beautiful color and appearance, are an important regional landscape resource in Xinjiang. The mountain flower communities formed by *Trollius* and their companion species are also important engines for maintaining the health of grassland and understory ecosystems in Xinjiang [35]. This study predicted the impacts of GCC on the evolution of suitable habitats and ecological niche changes for *Trollius* wildflowers in ITAP from the past to the future. The results indicated that precipitation and temperature are the dominant environmental variables affecting the distribution of *Trollius*. For *T. altaicus* and *T. lilacinus*, the precipitation factor was a deeper influence, potentially due to their adaptations to extreme temperatures in Xinjiang. These adaptations include entering dormancy or seeking shelter under extremely hot or cold conditions, which would allow them to survive despite these harsh conditions [36]. For instance, *T. altaicus* exhibits a capacity to dethatch its leaves and reduce its height, retaining only the number of leaves necessary for photosynthesis to survive the summer. In contrast, during the winter months, it enters a dormant state and withers, preparing for germination in the following spring. *T. lilacinus* displays greater tolerance to cold weather due to its prevalence at high altitudes. Nevertheless, both *T. altaicus* and *T. lilacinus* are highly susceptible to precipitation during the warmest season. In 2020, a field study was conducted in Xinjiang, where the climate was found to be abnormal, with increased temperature and decreased precipitation. This resulted in *T. altaicus* plants in Tacheng Bayimuzha and Urumqi South Tien Shan having low levels of growth, with smaller flower diameters and overall sub-healthy conditions compared to the previous wet year (Figure 9). This suggests that during the peak growing season of the plant, water and heat conditions are indispensable [37]. Previous large-scale species distribution prediction studies have not considered soil factors. However, soil physicochemical properties play a crucial role in species distribution. Simulation studies have shown that soil texture and pH are also among the environmental variables influencing the distribution of *Trollius*, particularly for *T. dschungaricus* and *T. lilacinus*. According to the research, *T. dschungaricus* is predominantly found in the understory of temperate mixed coniferous and broad forests, while *T. lilacinus* is found in alpine meadows, both of which are characterized by slightly acidic humus soils. Additionally, elevation and vegetation elements are also significant environmental variables influencing the distribution of *Trollius* wildflowers.

With greenhouse gas emissions, some species will migrate to higher latitudes or elevations, while others will maintain their current location through physiological or phenological changes [38]. The future climate scenario indicated that the changes in suitable habitat for *Trollius* wildflowers in the 2050s, 2070s, and 2090s exhibits uneven fluctuations. In general, the SSP1-2.6 scenario demonstrated a higher level of habitat maintenance. It was demonstrated that a certain degree of increased CO_2_ emissions could promote the growth of terrestrial plants and enhance photosynthetic efficiency [39]. This suggests that a moderate increase in CO_2_ concentration is favorable for the population expansion of *Trollius*, but a scenario of emissions that are too high may be detrimental to the survival of herbaceous plants because of their weaker carbon sequestration capacity than that of woody plants [40]. The establishment of the SSP3-7.0 scenario was associated with a decrease in future precipitation and an increase in temperature [24], yet the proportion of suitable habitat area of *T. asiaticus* remained consistently larger, which indicates that *T. asiaticus* is more susceptible to temperature changes. This result is consistent with a previous report by Li [41], which reveals an evolutionary bias in the response to environmental changes between the different species in the same genus. Furthermore, the transition zones between mountains and plains, such as hilly areas, are often the origin of increased habitats, and habitats are also the first to disappear in the transition zone. The edge theory indicates that the edge of an ecosystem often exhibits high biodiversity, yet it is inherently unstable and susceptible to external disturbances, particularly in recent times [42]. In the future, all four species of *Trollius* will have some degree of increased habitats in the Altay Mountains, which may be related to the increases in precipitation in the Altay Mountains in the future. Concerning Tacheng, the more arid climate results in plant communities being more sensitive to external random disturbances, which will lead to the disappearance of more habitats in the mountainous areas in Tacheng. Ili River Valley is characterized by a relatively humid climate and fertile soil and water resources, which provide a refuge for *Trollius* to cope with a range of extreme climatic conditions. It also serves as a concentrated distribution point for *Trollius*, but in the future, it may also face the problem of habitat degradation at the edge of the mountainous area, which needs to be prevented.

The climatic ecological niche is the ecological magnitude occupied by species in a multifactorial climate space [34]. The differentiation of climatic ecological niches not only represents the change in species distribution but also records the alteration in species’ dependence and occupancy on climatic elements. This is essentially the evolution of species due to genetic changes as a result of natural selection [43]. The ecological niches of *T. asiaticus* were predominantly expansive, occupying the potentially available space, indicating the spatial migration and resource-holding capacity of *T. asiaticus* when facing climate change, which can be also illustrated by the widest geographic distribution in China. However, considering the conservation of resource allocation, the expansion of *T. asiaticus* has the potential to affect other species in the community. The ecological niches of *T. lilacinus* were predominantly degraded, which revealed the vulnerability of alpine meadow ecosystems near the snow line. An overlap index greater than 0.9 can be considered to represent no differentiation. The results of this study indicate that the overlap indexes of *T. altaicus* are greater than 0.9 in some scenarios, suggesting a greater probability of stability. In contrast, the overlap indexes of the remaining three species range from 0.6 to 0.9, indicating a relatively large amount of potentially usable environmental space.

In addition to climate warming, the development of tourism, livestock, and illegal harvesting have also significantly reduced the habitat of *Trollius*. In recent years, the *Trollius* flower sea has become a popular tourist destination (the established species are mainly *T. altaicus* and *T. asiaticus*); however, the construction of scenic facilities and the influx of tourists have harmed the local populations of *Trollius*. Furthermore, the habitats of *Trollius* overlap with grazing land in Xinjiang due to a lack of targeted protection mechanisms, which results in the populations of *Trollius* being seriously trampled and gnawed by herds. The medicinal and food value of *Trollius* has made illegal harvesting a major factor in the sharp decline in *Trollius* populations. For example, *T. lilacinus*, growing near the snow line, has been subjected to severe poaching for its tea properties. As a result, *T. lilacinus* has been classified as a vulnerable (VU) species in China. Based on the simulation results of the existing and potential habitats of *Trollius*, it is strongly recommended that the wild resources of *Trollius* be protected holistically, taking into account the Xinjiang Territorial Spatial Planning Document (2021–2035) and the results of the Third Xinjiang Scientific Expedition. Firstly, it is essential to scientifically divide the source areas of *Trollius* habitat by combining the scope of existing nature reserves and highly suitable habitat distribution and establishing ecological corridors between the source areas for migration, for example, making lowly and moderately suitable habitats become stepping stones and connections for the migration process of *Trollius*. Secondly, building ecological barriers such as Tien Shan, Altay Mountains, etc., to construct a biodiversity conservation network, to strengthen the protection of natural forests and meadow in the native range of *Trollius*, to scientifically demarcate the ecological red line, and to limit the harm of tourism, livestock increases, and the expansion of construction on the *Trollius* population. Finally, it is necessary to implement protection regulations, strictly combat illegal poaching, and carry out artificial cultivation of *Trollius*.

In conclusion, this study systematically explored the impacts of environmental variables on *Trollius* wildflowers and changes in suitable habitats and ecological niches and made relevant recommendations for the future conservation of *Trollius*. However, additional studies still need to be enhanced. In the future, field research should be strengthened to obtain real-time distribution data and local climatic data. Additionally, concerning the selection of environmental factors, it would be beneficial to consider the inclusion of human activities and land-cover change factors in future studies. Furthermore, the core patches and potential corridors of suitable habitats for the species could be integrated with the nature reserves, thus forming a systematic habitat network that would be more conducive to the conservation of the rare species.

## 5. Conclusions

In this study, we employed the R-optimized MaxEnt model to predict the suitable habitat distribution of four *Trollius* species in the past, current, and future under multiple scenarios. Additionally, we identified the alteration in climatic niche breadth and overlap index based on environmental variables and suitable habitat distribution patterns. The following conclusions can be drawn.

(1) The climatic variable that most strongly influenced the distribution of *Trollius* is Bio18 (precipitation of warmest quarter), while Bio4 (temperature seasonality) has a wide effect on species’ distribution. Additionally, vegetation type and soil factors, which are determined by altitude, also contributed to the distribution of *Trollius*.
(2) At present, the proportion of suitable habitats for *Trollius* wildflowers varies from 20.46% to 40.68%. The suitable habitats of *Trollius* wildflowers are mainly distributed in high-altitude mountainous areas, with Ili River Valley and the Altay Mountains being the best distribution areas for *Trollius*.
(3) The percentage of suitable habitats for *Trollius* wildflowers varies greatly from the past to the future, ranging from 0.14% to 70.97%, and the area of preserved suitable habitat for *Trollius* is larger in the SSP1-2.6 scenario, whereas the four *Trollius* species differ in their adaptive capacity for the SSP3-7.0 and SSP5-8.5 scenarios. Suitable habitats are mainly at the edge of the mountainous region and migrate to higher latitudes.
(4) The evolution of ecological niches reveals the multidimensional adaptive capacity of the species to climate change. The niche change in *T. asiaticus* is dominated by expansion, reflecting tfhe species’ stronger climate strain and migration ability, while the niche of *T. lilacinus* is dominated by degradation, exposing the climatic vulnerability of alpine flowers. The probability of niche stability is higher in *T. altaicus*, while the presence of both expansion and degradation lie in the niche of *T. dschungaricus*.

In conclusion, *T. altaicus* and *T. asiaticus* demonstrate promising potential for development under future climate scenarios, while *T. dschungaricus* and *T. lilacinus* face unfavorable prospects. This study provides valuable scientific guidance for the conservation planning of *Trollius* wildflowers by understanding their habitats and identifying niche alterations to promote sustainable development and biodiversity conservation in Xinjiang.

## Figures and Tables

**Table 1:** Contribution rates and threshold ranges for environmental variables.

Species	Dominant Factors	Contribution/%	Threshold Value	Mean Value	Species	Dominant Factors	Contribution/%	Threshold Value	Mean Value
*T. altaicus*	Bio18	43.2	93.96–157.56 mm	125.76 mm	*T. dschungaricus*	T_PH_H_2_O	44.7	423, 964.56–553,077.70	488,521.13
	NDVI	24.4	150.14–240.11	195.13		Bio1	12.8	−3.92 to 3.94 °C	0.01 °C
	Bio6	9.4	−19.85 to −12.68 °C	−16.36 °C		NDVI	12.6	186.25–280.50	233.38
	Alt.	5.8	1170.81–2070.18 m	1620.50 m		Bio19	10.5	33.69–78.90 mm	56.3
	Bio4	4.8	1049.47–1229.15	1139.31		Bio4	7.6	789.71–1117.82	953.77
*T. asiaticus*	Bio3	37.4	27.08–38.76	32.92	*T. lilacinus*	T_PH_H_2_O	33.7	442,064.54–553,077.70	494,571.12
	Bio4	14.0	789.71–1221.34	1005.53		Bio18	19.0	174.35–281.20 mm	227.78 mm
	Bio17	9.1	21.60–78.90 mm	50.25 mm		Bio15	10.3	76.39–104.15	90.3
	Alt.	8.2	1269.52–2212.77 m	1741.14 m		T_USDA_TEX	5.9	442,064.53–5,553,077.70	497,571.11
	Bio5	7.7	0.73–26.31 °C	13.52 °C		Bio16	5.9	176.08–296.50 mm	236.29 mm
	Bio14	7.5	6.53–23 mm	14.77 mm		Bio4	4.8	789.71–1084.62	937.17
	NDVI	5.7	109.75–280.5	195.17		NDVI	4.2	161.16–280.50	220.83

**Table 2:** The area and proportion of suitable habitats for *Trollius* wildflowers in the current era.

Species	Highly Suitable Habitat		Moderately Suitable Habitat		Lowly Suitable Habitat		Unsuitable Habitat	
	Area/km^2^	Proportion/%	Area/km^2^	Proportion/%	Area/km^2^	Proportion/%	Area/km^2^	Proportion/%
*T. altaicus*	24,745.63	7.61	45,756.87	14.07	61,769.21	18.99	192,845.30	59.32
*T. asiaticus*	26,835.38	8.25	39,727.39	12.22	56,442.16	17.36	202,112.07	62.17
*T. dschungaricus*	16,660.84	5.12	29,325.41	9.02	44,572.94	13.71	234,557.82	72.15
*T. lilacinus*	18,783.73	5.78	17,957.19	5.52	29,788.07	9.16	258,588.02	79.54

**Table 3:** Environment variables involved in modeling.

Environmental Variable Types	Environmental Variable Description	*T. altaicus*	*T. asiaticus*	*T. dschungaricus*	*T. lilacinus*
Bioclimate variables	Annual Mean Temperature (BIO1)	E	E	E	E
	Mean Diurnal Temperature Range (BIO2)	E	E	E	E
	Isothermality (BIO3)	E	E	E	E
	Temperature Seasonality (BIO4)	E	E	E	E
	Max Temperature of Warmest Month (BIO5)		E		
	Min Temperature of Coldest Month (BIO6)	E	E	E	
	Temperature Annual Range (BIO7)	E			E
	Mean Temperature of Wettest Quarter (BIO8)			E	E
	Mean Temperature of Driest Quarter (BIO9)			E	
	Mean Temperature of Warmest Quarter (BIO10)	E			
	Mean Temperature of Coldest Quarter (BIO11)		E	E	
	Annual Precipitation (BIO12)	E		E	E
	Precipitation of Wettest Month (BIO13)	E	E		
	Precipitation of Driest Month (BIO14)	E	E	E	E
	Precipitation Seasonality (BIO15)	E	E	E	E
	Precipitation of Wettest Quarter (BIO16)	E		E	E
	Precipitation of Driest Quarter (BIO17)	E	E		
	Precipitation of Warmest Quarter (BIO18)	E	E	E	E
	Precipitation of Coldest Quarter (BIO19)	E		E	E
Topographic variables	Altitude (Alt.)	E	E	E	E
	Slope	E	E	E	E
	Aspect	E	E	E	E
	Vector ruggedness measure (VRM)	E	E	E	E
	Terrain roughness index (TRI)	E	E	E	E
Soil variables	Soil PH (T_PH_H_2_O)	E	E	E	E
	USDA soil texture classification (T_USDA_TEX)	E	E	E	E
Vegetation variables	Normalized difference vegetation index (NDVI)	E	E	E	E

Note: “E” means this environmental variable is elected.

**Table 4:** Results of the optimal parameter combination for the model.

Species	Omission Rate at 5%	Delta AICc	Best Model
*T. altaicus*	0.04	0	RM:3.5, FC: LH
*T. asiaticus*	0	0	RM:2.7, FC:PT
*T. dschungaricus*	0	0	RM:1.1, FC:Q
*T. lilacinus*	0	0	RM:3.5, FC: QP

## Data Availability

The data presented in this study are available in this article.

## Supplementary Materials

The following supporting information can be downloaded at: https://www.mdpi.com/article/10.3390/plants13131752/s1, Figure S1. SPSS analysis. Figure S2. PCA analysis of niche. Figure S3. Omission rate and AICc value. Table S1. Niche breadth value. Table S2. The area of suitable habitats. Table S3. The distance of gravity center transfer.
