# Supplementary figures and images for: Impacts of Climate Change on the Distribution of Suitable Habitats and Ecological Niche for Trollius Wildflowers in Ili River Valley, Tacheng, Altay Prefecture
Source: Plants (Basel). 2024 Jun 25;13(13):1752. doi: 10.3390/plants13131752 (PMC11243624; doi:10.3390/plants13131752)

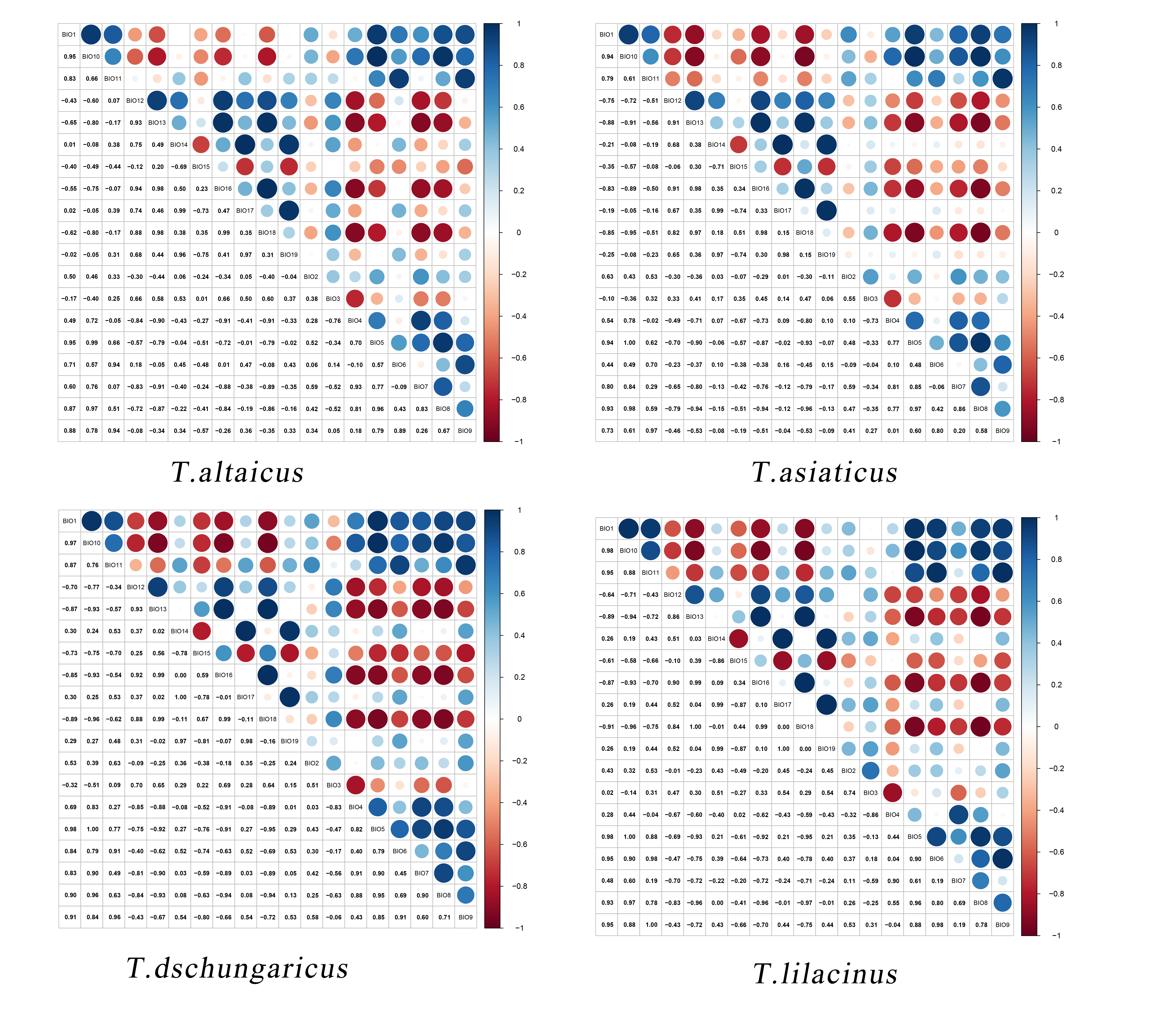

Supplement: Supplementary file 1 [file plants-13-01752-s001.zip › plants-3039319-supplementary/supplementary files/Figure_S1_spss analysis.png]

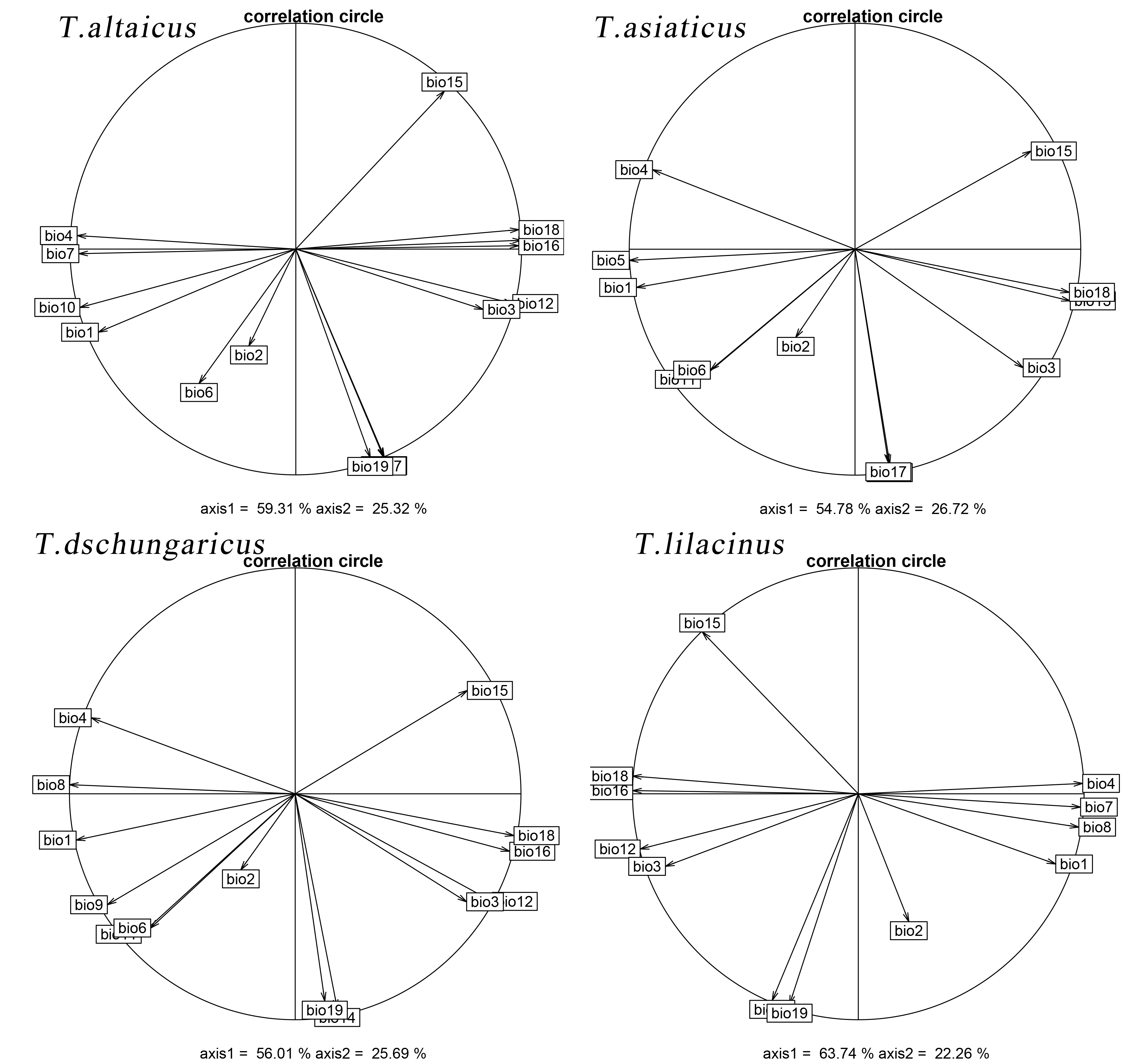

Supplement: Supplementary file 1 [file plants-13-01752-s001.zip › plants-3039319-supplementary/supplementary files/Figure_S2_PCA analysis of niche.png]

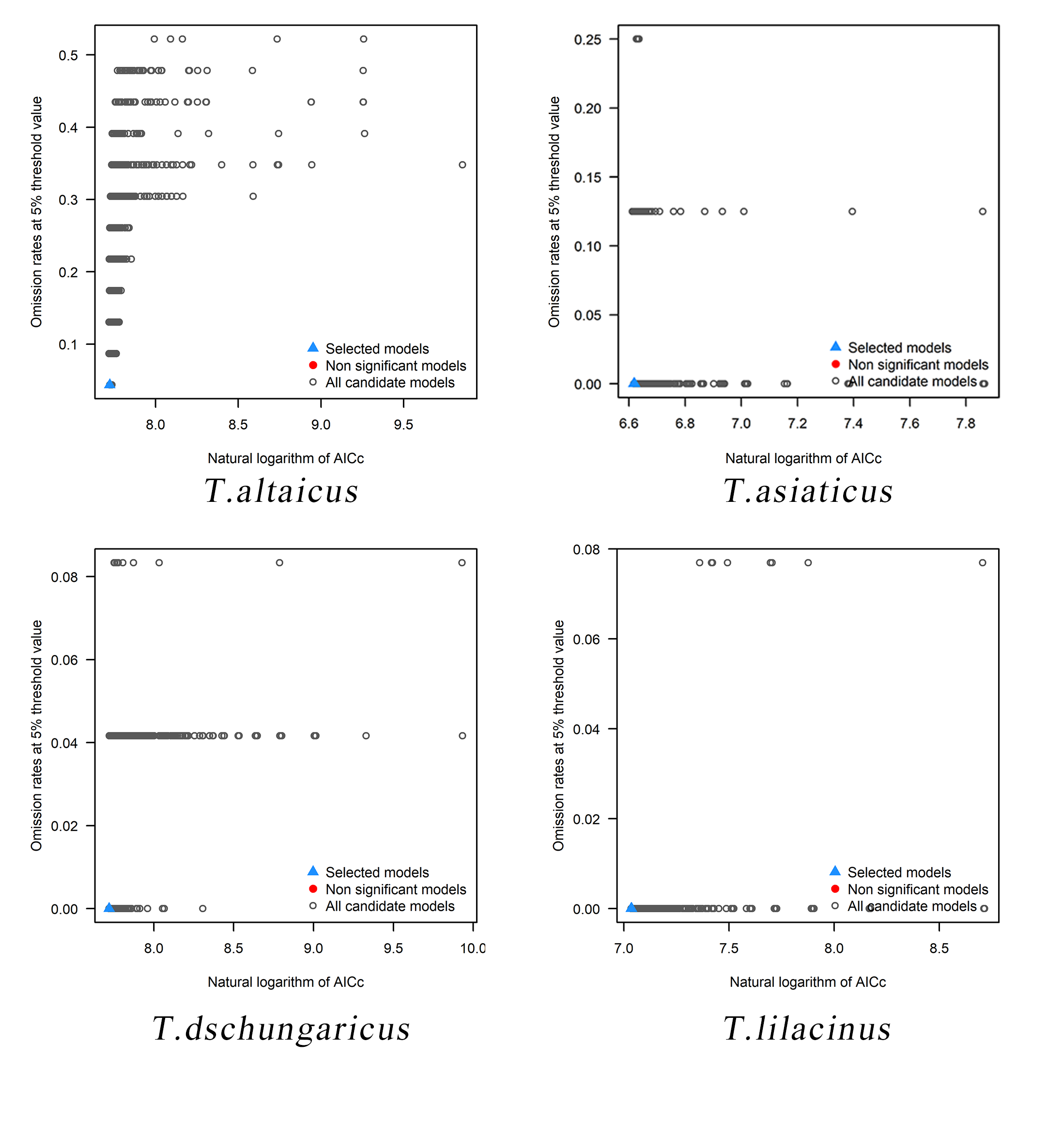

Supplement: Supplementary file 1 [file plants-13-01752-s001.zip › plants-3039319-supplementary/supplementary files/Figure_S3_omission rate and AICc value.png]
